# Global gridded GDP data set consistent with the shared socioeconomic pathways

**DOI:** 10.1038/s41597-022-01300-x

**Published:** 2022-05-19

**Authors:** Tingting Wang, Fubao Sun

**Affiliations:** 1 Key Laboratory of Water Cycle and Related Land Surface Processes, Institute of Geographic Sciences and Natural Resources Research, Chinese Academy of Sciences, Beijing, 100101 China; 2 State Key Laboratory of Desert and Oasis Ecology, Xinjiang Institute of Ecology and Geography, Chinese Academy of Sciences, Urumqi, 830011 China; 3 Akesu National Station of Observation and Research for Oasis Agro-ecosystem, Akesu, 843017 China; 4 College of Resources and Environment, University of Chinese Academy of Sciences, Beijing, 100049 China

**Keywords:** Climate change, Socioeconomic scenarios

## Abstract

The vulnerability, exposure and resilience of socioeconomic activities to future climate extremes call for high-resolution gridded GDP in climate change adaptation and mitigation research. While global socioeconomic projections are provided mainly at the national level, and downscaling approaches using nighttime light (NTL) images or gridded population data can increase the uncertainty due to limitations. Therefore, we adopt an NTL-population-based approach, which exhibits higher accuracy in socioeconomic disaggregation. Gross regional product of over 800 provinces, which covering over 60% of the global land surface and accounted for more than 80% of GDP in 2005, were used as input. We present a first set of comparable spatially explicit global gridded GDP projections with fine spatial resolutions of 30 arc-seconds and 0.25 arc-degrees for the historical period of 2005 and for 2030–2100 at 10-year intervals under the five SSPs, accounting for the two-child policy in China. This gridded GDP projection dataset can broaden the applicability of GDP data, the availability of which is necessary for socioeconomic and climate change research.

## Abstract (summary)

**Table:** 

Measurement(s)	GDP • population • nighttime light images
Technology Type(s)	SSP database • LandScan Global Population database • Remote sensing
Sample Characteristic - Location	Globe

## Background & Summary

The shared socioeconomic pathways (SSPs), which qualitatively and quantitatively describe possible future patterns of global socioeconomic development under different challenges to climate change mitigation and adaptation^1^, are of vital importance in global scientific assessment reports^2,3^ and the current literature^4,5^. The widely used Scenario Model Intercomparison Project (ScenarioMIP) was formed based on different SSPs corresponding to specific representative concentration pathways (RCPs) within Phase Six of the Coupled Model Intercomparison Project (CMIP6)^4^. Future socioeconomic impacts on climate change are built upon socioeconomic projections and require gridded gross domestic product (GDP) data of a higher resolution for future scenarios^4,5^.

GDP is the most commonly used indicator to assess and compare economic development within and across countries^6–8^. Some global institutions (e.g., the World Bank, the Organization for Economic Co-operation and Development (OECD)), research groups (e.g., Penn World Tables (PWTs)), and researchers^6,9^ have collected or extended global GDP data for various applications. However, gross regional product (GRP) data at the provincial (state), city or county scales are often problematic, especially in many developing countries^7,8^. Kummu *et al*.^7^ presented a set of global gridded GDP data in 2011 purchasing power parity (PPP) international dollars for 1990–2015 at 5 arc-minutes annually and at 30 arc-seconds for 1990, 2000, and 2015, making it easily integrated with data from other disciplines^10,11^. While national and supranational GDP in the SSP database, which is the most widely used inputs in disaggregation, are mainly in 2005 PPP USD, resulting in biased data when compared with other historical periods directly. It is therefore crucial to spatialize GDP in 2005 PPP USD at a fine scale so that it can be easily analyzed and compared with GDP projections of future scenarios.

Gridded GDP estimates can be obtained using a spatially explicit gridded population dataset and/or satellite-derived nighttime light (NTL) images to better support current research^8,12–15^. However, the GDP proportional to population estimates are based on the assumption that GDP per capita is uniformly distributed within an administrative boundary and are often biased at smaller scales^9^. The Defense Meteorological Satellite Program’s Operational Linescan System (DMSP-OLS) NTL images have been widely used in GDP downscaling for 1992–2013. However, these gridded GDP estimates are highly dependent on the NTL values and are often underestimated in urban centers and overestimated in rural regions due to the saturation problem^16^. Fortunately, this can be mitigated to a certain extent when incorporating gridded populations^17,18^. In contrast, the global Soumi National Polar-Orbiting Partnership Visible Infrared Imaging Radiometer Suite (NPP-VIIRS) NTL images address this saturation problem and provide a more accurate proxy for GDP downscaling after 2012^12^.

The increasing vulnerability, exposure, and resilience of socioeconomic activities to future climate extremes are promoting a move beyond administrative boundaries to enable flexible integration with the socioeconomic projections of the five SSPs^5,19,20^. However, the widely used GDP projections in the SSP database are restricted at national and supranational scales and depict a wide range of uncertainties within organizations^21,22^. The spatially explicit demographic projections at 0.125 arc-degrees^4^ have been further downscaled to 1-km resolution^23^ and are publicly available, but the GDP projections are rather limited in their ability to facilitate global climate change research. Using gridded population estimates, Geiger *et al*.^9^ downscaled and provided a set of spatially explicit global GDP estimates at 5 arc-seconds for 1850–2100 under SSP2 scenario. Murakami and Yamagata^24^ disaggregated the global population and GDP into 0.5 arc-degree grids for SSP1–3 scenarios, but SSP4-5 projections were unavailable to the public. Jiang *et al*.^25,26^ projected the Chinese population and GDP at the provincial level, and Huang *et al*.^27^ later showed that Chinese GDP would increase by 38.1–43.9% by the late 21st century under the two-child policy.

To date, high-resolution global gridded GDP projections for all five SSPs are rather limited, and historical GDP has been bound to various data sources and to clearly limited downscaling approaches that use population estimates or NTL images. Hence, we downscale and present a set of comparable spatially explicit global GDP estimates for both the historical period and 2030–2100 at 10-year intervals. These estimates have high spatial resolutions of 1 km and 0.25 arc-degrees under all five SSPs, obtained by incorporating reliable data sources and methods to broaden the applicability of GDP data to scenario-based climate change research.

## Data and Method

**Fig. 1:**
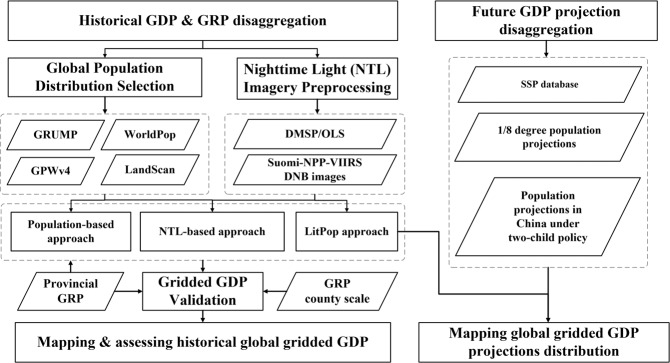
Workflow for developing the spatially explicit GDP dataset consistent with the SSPs.

The NTL images and gridded population estimates are well correlated with GDP and have been widely used in global and regional GDP disaggregation at various spatial scales but have their own limitations, as described above. The pursuit of a reliable GDP projection dataset requires more accurate inputs, proper downscaling approaches, etc. In what follows, we describe the data, including the gridded population estimates and NTL images used to generate the base map, GDP and GRP, and the methods used for GDP downscaling. The data cleaning and estimation workflow is presented in Fig. 1.

### Gridded population dataset

Gridded population data have been widely used to spatialize GDP (GRP) to a fine scale^9,18^. To obtain a set of highly accurate global gridded population data, data from the Gridded Population of the World dataset, Version 4 (GPWv4), Revision 11 (http://sedac.ciesin.columbia.edu/data/collection/gpw-v4)^28^, WorldPop (www.worldpop.org)^29,30^, the Global Rural-Urban Mapping Project, Version 1 (GRUMPv1) dataset (https://sedac.ciesin.columbia.edu/data/set/grump-v1-population-density), and the LandScan Global Population database (https://landscan.ornl.gov/)^31^ are compared at the national, provincial (state), and county scales first (Figures S1, S2). The LandScan Global Population database from the Urban Oak Ridge National Laboratory, USA, was chosen due to its relatively higher accuracy at the county scale (Figure S2), its best census and geographic data in terms of availability, and remote sensing imagery analysis techniques within a multivariate dasymetric modeling framework to disaggregate census counts within an administrative boundary^31^.

### Nighttime light images

The NTL images are well correlated with global and regional socioeconomic activities and have also been widely used in GDP disaggregation^8,14^. The widely used version 4 of the DMSP-OLS stable NTL images in 2005 and 2012 were obtained from the National Oceanic and Atmospheric Administration’s National Geophysical Data Center (NGDC) at https://www.ngdc.noaa.gov/eog/dmsp/downloadV4composites.html. These data have a spatial resolution of 30 arc-seconds and latitudinal and longitudinal ranges of 75°N to 65°S and 180°W to 180°E. The values for the DMSP-OLS stable NTL data range from 0 to 63, and the saturation problem (value of 63) is significant, mainly in large city centers and other brightly lit zones^12^.

In addition, the Suomi-NPP-VIIRS Day/Night Band (DNB) images (https://eogdata.mines.edu/download_dnb_composites.html) from 2015 were obtained. These images have a higher resolution of 15 arc-seconds. To ensure the same cell size, NPP-VIIRS NTL images were resampled to a spatial resolution of 30 arc-seconds. In addition, the DMSP-OLS images in 2012 were used to eliminate the negative values in the NPP-VIIRS DNB images.

### GDP and GRP

The World Development Indicators assembled by the World Bank (WB-WDI) provide a vast resource of relevant, high-quality, and internationally comparable socioeconomic statistics for 217 economies from 1961 to the present. For most economies, GDP in 2017 PPP international dollars, in current prices, and in other units can be easily obtained from https://databank.worldbank.org/home.aspx. However, GDP in 2005 PPP USD is no longer available from the WB-WDI, which makes GDP projections under the SSPs in 2005 PPP USD incomparable with historical data. We therefore obtained the GDP of 195 countries from Geiger *et al*.^6,9^, which mainly comes from PWT8.1, in 2005 PPP USD, which is consistent with the SSP projections, and data for missing countries are taken from PWT9.0 and the WB-WDI after rescaling from 2011 to 2005 PPP USD.

GRP in current PPP USD for over 800 provinces (states) in 48 countries (35 OECD countries plus 13 non-OECD countries) were obtained from OECD data at https://stats.oecd.org/Index.aspx?DatasetCode=SNA_TABLE1. However, GRP data for Egypt (http://hdr.undp.org/sites/default/files/2008_egypt_nhdr_en.pdf) and Kenya (http://hdr.undp.org/sites/default/files/kenya_2006_en.pdf) were found in the Human Development Reports from the United Nations Development Programme since GRP (PPP) and GRP per capita (PPP) in 2005 were provided. These 50 countries cover over 60% of the global land surface and accounted for more than 80% of global GDP in 2005.

Moreover, GRP at a more explicit county-level was obtained for China (2160 counties with valid data in 2005 from the China County Statistical Yearbook 2006) and the U.S. (3071 out of 3145 counties from the U.S. Bureau of Economic Analysis (https://www.bea.gov/data)) for validation purposes. The Chinese figures were converted to USD using the conversion factors provided by the World Bank.

### SSP projection data

Long-term demographic and GDP projections have been made available by different organizations to facilitate research on future impacts, adaptation, and vulnerability. There are several widely used demographic and GDP projections available to the public.

#### SSP database

Based on the assumptions of the SSP storylines in terms of the main drivers of economic growth, three sets of global GDP projections have been developed and are provided in the SSP database^21,32–34^. As recommended by the SSP database, GDP projections in 2005 PPP USD were obtained from the OECD^33^ for 184 countries up to the end of the 21st century under the five SSP scenarios at https://tntcat.iiasa.ac.at/SspDb. Then, national GDP growth rate projections relative to 2005 can be calculated using these figures. Projections in the remaining missing countries were filled in by using supranational GDP growth rate projections.

The national and supranational population projections in the SSP database fail to meet the increasing demand for spatially explicit demographic projections for these future scenarios. Hence, we obtained the gridded population projections developed by Jones and O’Neill^4^, who further extended these national totals and produced a scenario-based gridded population dataset that is qualitatively consistent with the assumptions in the SSP narratives regarding spatial development patterns at a spatial resolution of 1/8° (approximately 7.5 arc-minutes at the equator). The GRUMP population dataset from 2000 was used as the base map. Using a parameterized gravity-based downscaling model, the demographic drivers, namely, current fertility, income, urbanization, and international migration, are explicitly included in the population projections corresponding to each SSP to produce gridded population projections for all five SSPs that are quantitatively consistent with national population and urbanization projections.

#### Chinese population projections under the two-child policy

The implementation of the two-child policy in 2016 will no doubt have a substantial effect on the demographic and socioeconomic projections for China in the long run. In addition, the clear differences among the drivers (e.g., population distribution by age, sex, and educational enrollment in the historical period) between the census data in China and the United Nations (UN) estimations^27^ require revisions and updates to the Chinese population and GDP projections for the five SSPs. Jiang *et al*.^25,26^ employed data from the China Statistical Yearbook and the Sixth National Population Census and made projections for 2020–2100 based on assumptions about future education, fertility, mortality, and migration for the five SSPs. Their projections account for the two-child policy. Using a parameterized multidimensional model, population projections by age, sex, education, and the economy are developed for China up to the year 2100 under the framework of the SSPs but are revised based on regional characteristics. The provincial demographic projections are further downscaled and provided at a 0.5-degree resolution for the five SSPs.

### Population-based GDP disaggregation method

A broad literature has emphasized the key role of human capital as a driver of socioeconomic growth and population is the central link between human capital and economic growth in almost all econometric models^32,33,35^. Shiogama *et al*.^36^ suggested the robustness of an ensemble learning-based downscaling approach, which is defined by (baseline variable) × (control variable) in accordance with distribution weights. GDP can thus be proportionally disaggregated to match population counts based on the assumption that GDP per capita is uniformly distributed within a given administrative boundary. This population-based GDP disaggregation (denoted GDP_pop_) can be described as follows:

1 $$GD{P}_{Pop}=Po{p}_{pixel}\times Pca{p}_{i}=Po{p}_{pixel}\times \frac{GD{P}_{i}}{Po{p}_{i}}$$

where Pop_pixel_ is the population count in each grid, which acts as the baseline variable, and Pcap_i_ (GDP per capita within a given administrative boundary i) acts as the control variable.

### NTL-based GDP disaggregation method

NTL images have served as a useful proxy for socioeconomic development and have been widely used to improve the quality of socioeconomic data, especially in many developing countries given their high spatial resolution^8,14,16^. There are two separate annual stable light images derived from two satellites (F14 and F15, working simultaneously to collect global NTL information) in DMSP-OLS, and average visible, stable light, and cloud free coverage products are provided for 2005. The stable light product from F15, which contains a composite of all available cloud-free data with a spatial resolution of 1 km, was chosen since newer sensors have less degradation in their data quality and have been utilized to disaggregate global GDP into 1 km × 1 km grids. The NPP-VIIRS products have several demonstrated improvements over the DMSP-OLS NTL images, e.g., resolving the saturation problem and improving their calibration with a higher spatial resolution of 15 arc-seconds. Given their public availability and regular updates, global NTL images from two satellites have proven to be a useful source of information and are the most commonly used proxy in socioeconomic and climate change research. Based on the statistical linear relationship between GDP and the luminosity in NTL images, GDP is directly redistributed to each grid in proportion to the values within a given administrative boundary. Thus, the NTL-based GDP (denoted GDP_Lit_) can be described as:

2 $$GD{P}_{Lit}=GD{P}_{per\_light}\times Li{t}_{pixel}=\frac{GD{P}_{i}}{S{L}_{i}}\times Li{t}_{pixel}$$

where GDP_i_ is a GDP total, SL_i_ is the sum of the values from NTL images of administrative unit i, and GDP_per_light_ is a constant within this administrative unit i.

### NTL-population-based approach

Studies have shown that the saturation problem in the DMSP-OLS NTL images has resulted in overestimation for large urban areas and underestimation for rural and distant regions^16–18^. Zhao *et al*.^18^ improved the accuracy of GDP disaggregation by combining NTL images with gridded population data using a function in which GDP is proportional to NTL luminosity and population count and named it the LitPop approach. Eberenz *et al*.^17^ further improved this method by filling in the nonilluminated cells with population counts and the depopulated zones with NTL values, providing a better proxy for the spatial disaggregation of economic indicators. The LitPop images were first produced by multiplying the data from the NTL images with data from the Landscan population dataset (Eq. 3), assuming that the values in the nonilluminated grids are equal to 1 to ensure that nonilluminated but populated grid cells are not assigned a value of zero and that illuminated but unpopulated areas are not assigned a value other than zero. The LitPop images were then used (Eq. 4) for GDP downscaling (denoted GDP_LitPop_) across the globe.

3 $$LitPop=\left\{\begin{array}{ll}Pop & Lit=0\\ Lit\cdot Pop & Lit > 0\& Pop > 0\\ Lit & Pop=0\end{array}\right.$$

4 $$GD{P}_{LitPop}=\frac{GD{P}_{i}}{SL{P}_{i}}\times {{\rm{LitPop}}}_{pixel}$$

where LitPop is the value of each pixel in the LitPop images, and SLP_i_ is the sum of the LitPop data within administrative unit i.

### Global GDP downscaling in 2005

Since GRP estimates in 2005 PPP USD are not available, we first made the following adjustments that are consistent with the SSP projections to ensure a more accurate GDP disaggregation. Using the ratio between GDP in 2005 PPP USD^6,9^ and GDP in current PPP USD (obtained from the WB-WDI) for each country, we first recalculated GRP in 2005 PPP USD based on a simple method (Eq. 5) under the assumption that the GDP in 2005 PPP USD is directly redistributed to each subnational area in proportion to the GRP in current prices within a given national administrative boundary i, overlooking regional differences in inflation.

5 $$GR{P}_{2005}=\frac{GD{P}_{i\_2005}}{GD{P}_{i\_current}}\times {{\rm{GRP}}}_{current}$$

GDP in 2005 PPP USD for the 50 countries mentioned above was replaced by the GRP of over 800 provinces (states) after being converted to 2005 PPP USD. Following the LitPop procedure, the NTL-based approach, and the population-based disaggregation method, 2005 GDP and GRP (in 2005 PPP USD) for countries across the globe were spatially linked to the corresponding GIS-based administrative boundaries (https://www.diva-gis.org/) and then disaggregated into 1 km×1 km grids using the DMSP-OLS NTL images and the Landscan population dataset for 2005. After making the comparison reported in the Technical Validation Section, the LitPop approach to GDP downscaling was adopted for both 2005 and for the future scenarios.

### GDP projection downscaling for the five SSPs

Using the LitPop approach, the GDP projections for the five SSPs were downscaled to a 1-km resolution. The GDP projections were first completed for all countries (regions) for the five SSPs. National projections for 177 countries and supranational projections for the world regions where the remaining countries are located were obtained from the OECD projections in the SSP database. GDP growth rates relative to 2005 (the figures in the SSP database) were calculated, and the supranational values were used to fill data for countries with missing values. GDP and GRP projections were then generated using these GDP growth rates and official figures from 2005 to complete the data series and ensure global consistency for all five SSPs.

Next, the population projections were downscaled to a 1-km resolution. Using the spatial distribution pattern in the Landscan population dataset for 2015, the 1/8-degree population projections consistent with the SSPs^19^ and the Chinese projections developed by Jiang *et al*.^25^ in the context of China’s two-child policy were downscaled to 1-km grids while still keeping the population totals at the original resolution. The two population projections were mosaiced together to create a new population raster for 2030–2100 at 10-year intervals for the five SSPs.

Finally, the global GDP projections were downscaled to a spatial resolution of 1 km using the LitPop approach. First, GDP and GRP projections were adopted and spatially linked to the corresponding administrative boundaries for 2030–2100 under the five SSPs. Second, the NPP-VIIRS DNB products for 2015 were used as the fixed NTL images, and the population projections that were downscaled to a 1-km resolution were used to generate the LitPop images for 2030–2100 under the five SSPs. Then, the GDP and GRP projections were disaggregated to a spatial resolution of 30 arc-seconds (~1 km) for all five SSPs.

In addition, the above spatially explicit GDP data for 2005 and 2030–2100 under the five SSPs were upscaled to 0.25 arc-degrees as an alternative choice.

## Data Records

In this research, we produced a set of comparable spatially explicit global GDP estimates, which, to the best of our knowledge, is the first dataset to present substantial long-term changes in global GDP (in 2005 PPP USD) between the historical period (with 2005 as a representative) and 2030–2100 under all five SSPs while accounting for the two-child policy in China^37^. This global gridded GDP dataset is provided in “GeoTIFF” format with two spatial resolutions: 30 arc-seconds and 0.25 arc-degrees. Global GDP is disaggregated within administrative boundaries, and therefore, Antarctica, oceans and some desert or wilderness areas (nonilluminated and depopulated zones) are assigned a value of 0. The spatial ranges are 60°S-90°N and 180°E-180°W in the standard WGS84 coordinate system. All files are available at 10.5281/zenodo.5880037^37^.

**Fig. 2:**
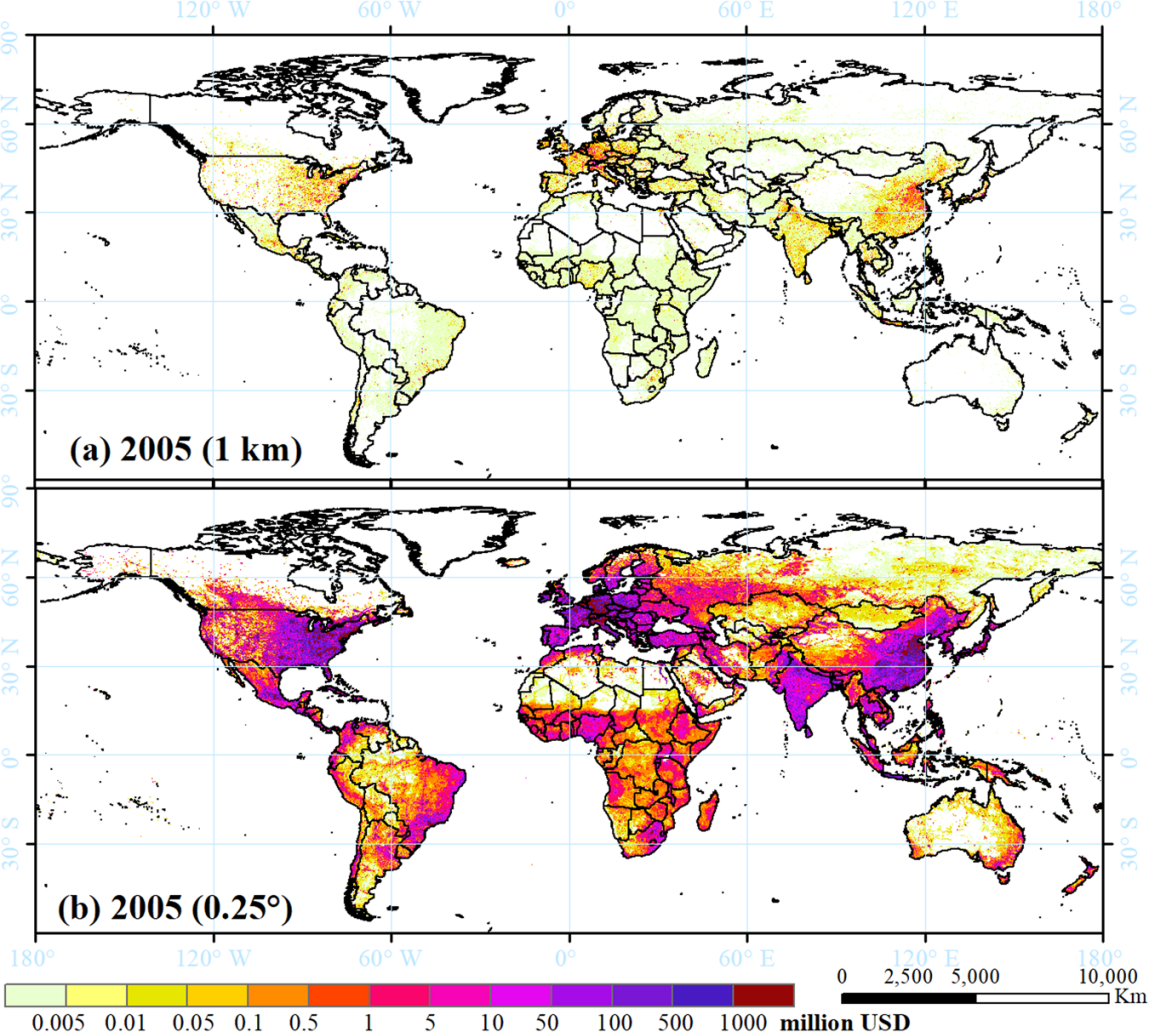
Spatial distribution of GDP in 2005 at spatial resolutions of 1 km (**a**) and 0.25 arc-degrees (**b**). The units are 2005 PPP USD per cell.

**Fig. 3:**
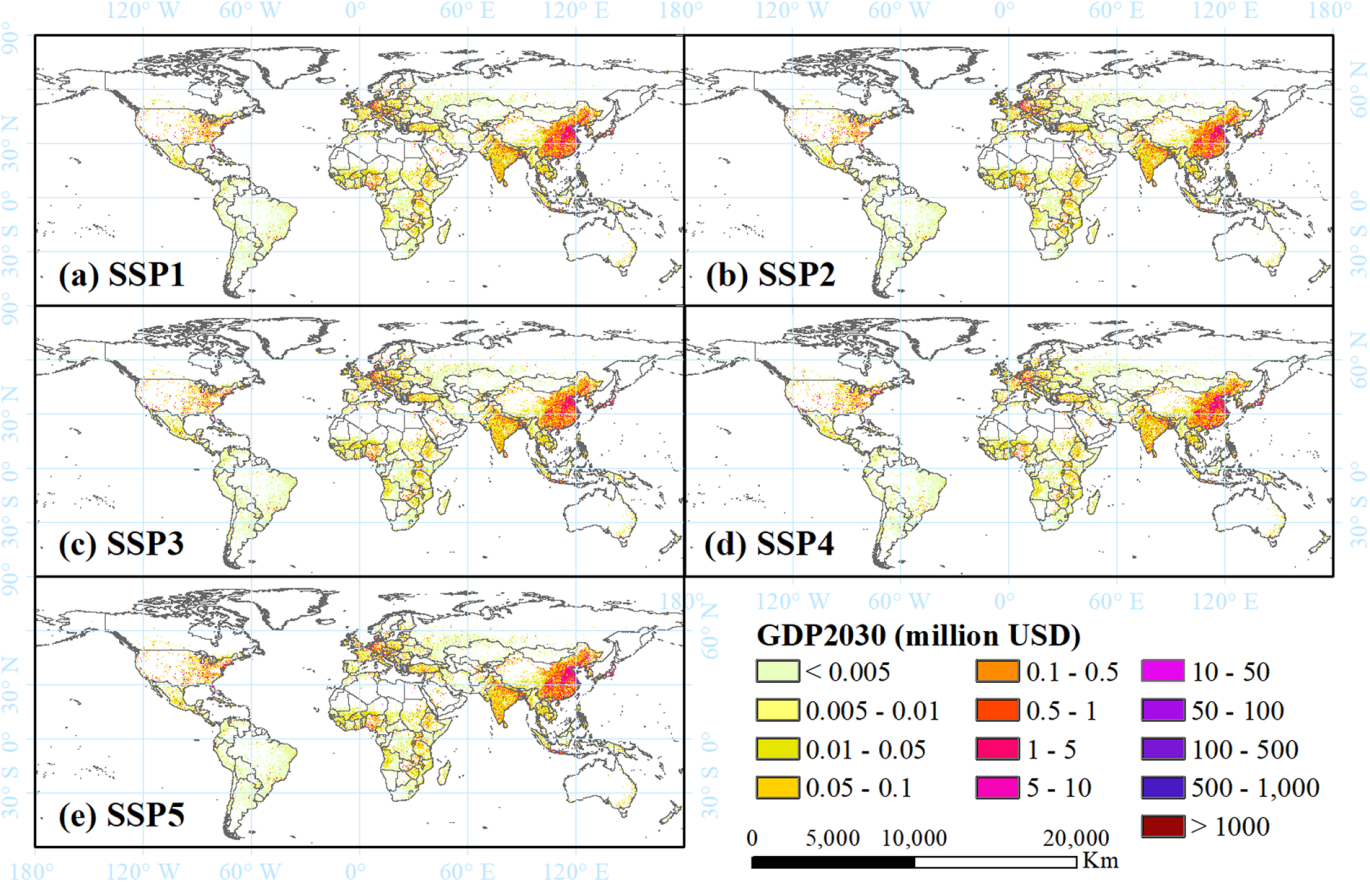
Spatial distribution of global GDP in 2030 under scenarios SSP1–5 (**a**–**e**) at a spatial resolution of 1 km. The units are 2005 PPP USD per cell.

The downscaled GDP for 2005 in 1-km and 0.25 arc-degree cells are shown in Fig. 2, and the GDP projections are shown in Fig. 3 for 2030 under SSP1-5 as a case study. This gridded GDP dataset can broaden the applicability of GDP data to allow for comparisons of global and regional socioeconomic changes between the historical period and future projections under the different SSPs. It can also increase research on scenario-based climate change and across disciplines.

## Technical Validation

To examine the performance of the three GDP disaggregation approaches described above, namely, GDP_Pop_, GDP_Lit_, and GDP_LitPop_, we first used national 2005 GDP for 203 countries only and spatialized the data into 1 km × 1 km grids based on each of the three approaches. The GRPs for over 800 provinces (states) in 50 countries and 5231 counties (in the USA and China) were used to evaluate these approaches by assessing their ability to disaggregate national GDP to the subnational level.

**Fig. 4:**
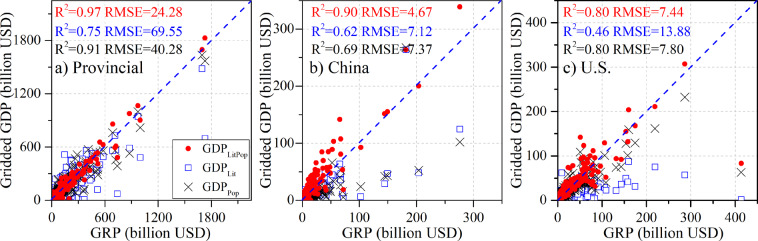
Evaluation of GRP and gridded GDP in 2005 (national GDP-based downscaling) at the provincial (state) (**a**) and county scales in China (**b**) and the U.S. (**c**) using the population-based disaggregation method, the NTL-based approach, and the LitPop approach.

The national comparison yields a high degree of accuracy, with all R^2^ values approaching 1.0 and very small RMSE values, which is to be expected since the national totals are inputs in each of these three methods. At the provincial (state) and county scales, the performance of GDP_LitPop_ is superior to that of GDP_Pop_ and GDP_Lit,_ with clear advantages in terms of slope, R^2^ and RMSE. Specifically, GDP_LitPop_ is more accurate in identifying the spatial allocation of GDP at a fine scale, with R^2^ values reaching 0.97, 0.90, and 0.80 and RMSE values of 24.28 billion, 4.67 billion, and 7.44 billion at the provincial (state) scale across the globe (Fig. 4a) and at the county scale for China (Fig. 4b) and the U.S. (Fig. 4c), respectively. However, the R^2^ values are only 0.75, 0.62, and 0.46, and RMSE values reach as high as 69.55 billion, 7.12 billion, and 13.88 billion, respectively, when using the GDP_Lit_ approach, exhibiting relatively large bias due to the saturation problem in the DMSP-OLS NTL images. In addition, GDP_Pop_ also identifies GDP redistributions at a fine scale well, with R^2^ and RMSE values close to but less accurate than those of GDP_LitPop_. It should be mentioned that the GRP values are in current prices, which would lead to additional bias if used directly or converted roughly to 2005 PPP USD.

The NTL images from NPP-VIIRS instead of those from DMSP-OLS in 2015 and the Landscan population dataset are used for the disaggregation of future GDP projections. To assess the performance of each approach in GDP downscaling, GRP figures for 737 provinces (states) and 2270 counties in China and 2996 counties in the U.S. from 2015 were obtained and used as another case for technical validation. The national GDP in 2015 in 2011 PPP international dollars for 199 countries from the WB-WDI were used for the GDP downscaling and then reaggregated into gridded GDP estimates at the provincial and county scales.

**Fig. 5:**
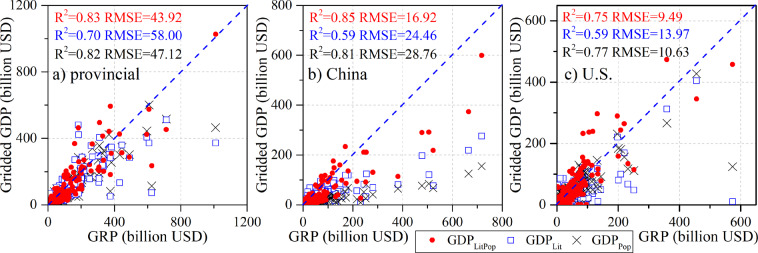
Evaluation of GRP and gridded GDP in 2015 (national GDP-based downscaling with data from the WB-WDI) at the provincial (state) (**a**) and county scales in China (**b**) and the U.S. (**c**), using the population-based disaggregation method, the NTL-based approach, and the LitPop approach.

Similar results to those for 2005 can be obtained for 2015 (Fig. 5). The comparisons show that GDP_LitPop_ outperforms the other methods with slightly higher R^2^ values of 0.83, 0.85, and 0.75 at the provincial (Fig. 5a), and county scales in China (Fig. 5b) and the U.S. (Fig. 5c). The RMSE values are thus the smallest at 43.92 billion, 16.92 billion, and 9.49 billion when compared with those for GDP_Lit_ (58.00 billion, 24.46 billion, and 13.97 billion) and GDP_Pop_ (47.12 billion, 28.76 billion, and 10.63 billion). This not only shows the superiority of the LitPop approach for GDP downscaling but also ensures the availability of gridded GDP projections by including the NPP-VIIRS NTL images from 2015 when building the LitPop base map for future SSPs.

**Fig. 6:**
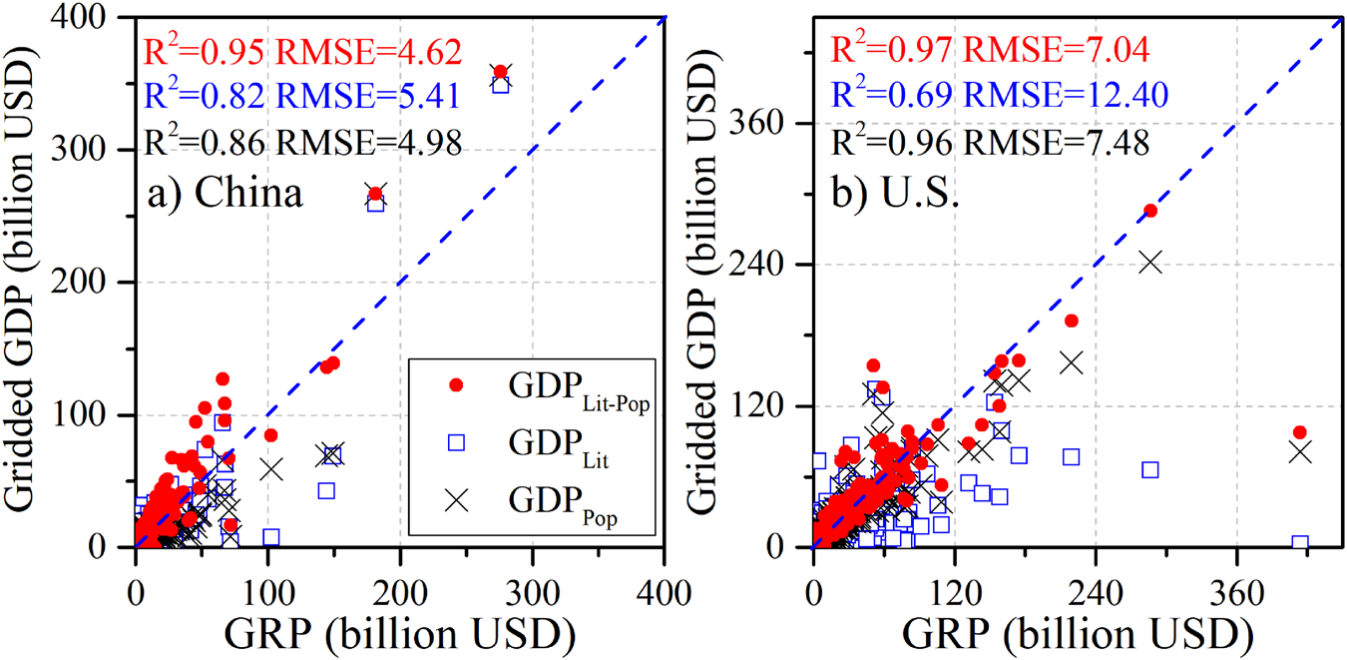
Evaluation of GRP and gridded GDP in 2005 (provincial GRP-based downscaling) at the county scale in China (**a**) and the U.S. (**b**), using the population-based disaggregation method, the NTL-based approach, and the LitPop approach.

We further assessed the performance of our gridded GDP estimates for 2005 by incorporating over 800 provincial (state) values into the downscaling procedure (Fig. 6). Estimates were obtained from the LitPop approach, the NTL-based approach, and the population-based disaggregation method. The performance of GDP_LitPop_ exceeded that of other two, with higher R^2^ values of 0.95 and 0.97 for China and the U.S., respectively. In addition, the RMSE values were smaller of 4.62 billion and 7.04 billion in China and the U.S., compared with the values of 5.41 billion and 12.40 billion for GDP_Lit_ and 4.98 billion and 7.48 billion for GDP_Pop_ (Fig. 6). This again shows that GDP_LitPop_ better identifies GDP redistributions at a fine scale. Moreover, the gridded GDP estimates at the provincial (state) scales are clearly excellent, with all R^2^ values approximately equal to 1.0, and the RMSE values decrease drastically to 0.14 billion, 0.32 billion, and 0.13 billion USD, respectively, indicating high accuracy when these provincial (state) GRP data are incorporated into the GDP downscaling. Additionally, the accuracy of the gridded GDP estimates at the county scale in China (Figs. 4b, 6a) and the U.S. (Figs. 4c, 6b) is also improved with much higher R^2^ values and smaller RMSE values for all three downscaling methods. This shows the high accuracy and credibility of both the LitPop approach and our gridded GDP dataset.

**Fig. 7:**
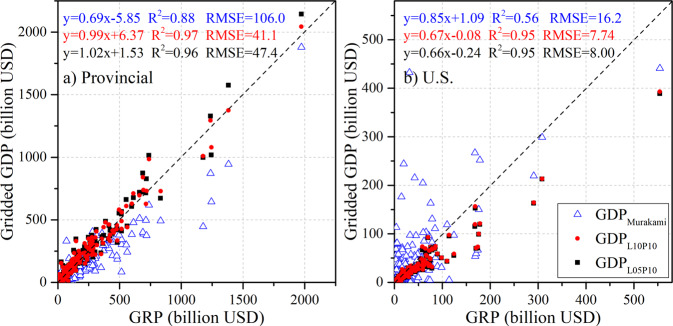
Evaluation of the GRP and gridded GDP from Murakami and Yamagata^24^ (denoted GDP_Murakami_) and national GDP disaggregation using the LitPop approach (two LitPop images: the NTL image in 2005 and the Landscan population in 2010 (denoted GDP_L05P10_) and the NTL image in 2010 and population in 2010 (denoted GDP_L10P10_)) at the provincial (state) scale (**a**) and at the county scale in the U.S. (**b**) in 2010.

We also compared and assessed the performance of the gridded GDP datasets from Murakami and Yamagata^24^, which include GDP estimates for 1980–2100 at 10-year intervals, through two scenarios using the LitPop approach for 2010 (Fig. 7). National GDP data from the WB-WDI were obtained and downscaled into 1-km grids using the LitPop approach. LandScan population data for 2010 and NTL images for 2010 and 2005 were used to generate two LitPop images: one with 2010 population and NTL images as a historical case (denoted GDP_L10P10_), and another with 2010 population and 2005 NTL images (denoted GDP_L05P10_) as a specious future scenario since fixed NTL images were used for the SSPs. Comparisons between GRP and gridded GDP in 304 provinces (states) (Fig. 7a) and 1996 counties with valid figures in each gridded dataset for the U.S. (Fig. 7b) show the superiority of our gridded GDP using the LitPop approach compared with that of Murakami and Yamagata^24^. The R^2^ values are much higher (over 0.95 vs. 0.56 at the county scale), and the RMSE values are approximately half as large (less than 50 billion vs. 106 billion USD at the provincial scale and approximately 8 billion vs. 16.2 billion at the county scale in the U.S.) (Fig. 7). This comparison also indicates that using NTL images from the historical period can induce additional bias in the future scenarios, and societal development could intensify the spatial differences across grids.

Above all, the LitPop approach has clear advantages for GDP disaggregation, especially at finer spatial scales, when compared with the population-based or NTL-based approaches. This was in line with many studies on overcoming the limitations of using either gridded population estimates based on the assumption of a uniform GDP per capita distribution across a given administrative unit or DMSP-OLS NTL images, which are subject to saturation problems^8,17,18,38^. Our gridded GDP dataset is recommended for use across disciplines due to its relatively high accuracy and credibility.

## Usage Notes

We adopted the LitPop approach and presented a first set of comparable spatially explicit global gridded GDP estimates with a high spatial resolution of 30 arc-seconds (approximately 1 km at the equator) as well as of 0.25 arc-degrees for 2005 and for 2030–2100 (in 2005 PPP USD) at 10-year intervals for all five SSPs, while accounting for the two-child policy in China. This gridded GDP projection dataset can broaden the ability of GDP data to account for the reality of global and regional socioeconomic changes between the historical period and future projections under the different SSPs. It can also increase research into scenario-based climate change.

To be clear, this GDP dataset is bound to input data from various sources and to the approaches used, including the use of a uniform GDP growth rate within administrative boundaries, the use of NPP-VIIRS data from 2015 as a fixed NTL image, and the use of the population distribution patterns from LandScan as a base map for future population downscaling. Moreover, the disaggregated GDP values may be underestimated in some underdeveloped areas where nonilluminated cells or unpopulated zones are located when using the LitPop approach. Therefore, these GDP projections fail to capture the spatial variation caused by migration and urban development in the future scenarios.

In addition, the GDP projections with a 1-km resolution are produced based on the assumption that there is no population mobility across grids when disaggregating the population projections from 1/8 degree^19^ to 1-km grids and then using them to build a future LitPop base map. In addition, the national and supranational GDP projections from the SSP database contain large amounts of uncertainty, as explained in Riahi *et al*.^21^, and should be treated with caution. The effects of financial crises, policy changes, technological progress, political and societal factors, climate system feedback, etc., are also not considered in this gridded GDP dataset.

## Supplementary information

Supplementary Information

## Data Availability

No specific code was generated for analysis of these data.
