# Supplementary material for: Global gridded GDP data set consistent with the shared socioeconomic pathways
Source: Sci Data. 2022 May 19;9:221. doi: 10.1038/s41597-022-01300-x (PMC9120090; doi:10.1038/s41597-022-01300-x)
Supplement: Supplementary file 1 — Supplementary Information [file 41597_2022_1300_MOESM1_ESM.docx]

Supporting Online Materials

**Global gridded GDP data set consistent with the shared socioeconomic pathways**

Tingting Wang, Fubao Sun^*^

# Supporting Figures





Figure S1 Comparisons between national (a) and provincial (b) population totals between official and gridded datasets in 2005, values in the legend are their corresponding RMSE.





Figure S2 Comparisons between official and gridded extractions from global population data sets for years of 2000 (a), 2005 (b), 2010 (c), and 2015 (d) at county level in China.
